# CRISPR-Based Tools for Fighting Rare Diseases

**DOI:** 10.3390/life12121968

**Published:** 2022-11-24

**Authors:** Qingyang Li, Yanmin Gao, Haifeng Wang

**Affiliations:** 1 Tsinghua-Peking Center for Life Sciences, School of Life Sciences, Tsinghua University, Beijing 100084, China; 2 Center for Synthetic and Systems Biology, Tsinghua University, Beijing 100084, China

**Keywords:** CRISPR-Cas, gene therapy, delivery system, rare disease

## Abstract

Rare diseases affect the life of a tremendous number of people globally. The CRISPR-Cas system emerged as a powerful genome engineering tool and has facilitated the comprehension of the mechanism and development of therapies for rare diseases. This review focuses on current efforts to develop the CRISPR-based toolbox for various rare disease therapy applications and compares the pros and cons of different tools and delivery methods. We further discuss the therapeutic applications of CRISPR-based tools for fighting different rare diseases.

## 1. Introduction

Rare diseases are routinely defined as those affecting a limited portion of the population, with slightly different prevalence thresholds around the world [1]. Despite the low prevalence of individual rare diseases, there are more than 7000 defined rare diseases affecting 3.5–5.9% of the global population (263–446 million people) [2]. Rare diseases are mostly chronic and genetically inheritable [3,4]. Symptoms commonly emerge early during patients’ childhood, leading to enormous physical and emotional pain and even premature death. Collateral suffering falls upon the family, leading to defective life quality and tremendous financial pressure. Patients are struggling for accurate diagnoses and efficient treatment, especially those in low-resource regions [5].

Gene therapy has brought promises for fighting rare diseases, given that approximately 80% of rare diseases are caused by genetic mutation and many of them have prenatal onset [6,7]. Compared to the previously approved therapies for rare diseases that relieve the symptoms using chemical agents, gene therapy targets the essential causes of rare diseases and provides optimized disease prognosis. CRISPR (clustered regularly interspaced short palindromic repeats)-based gene therapy tools display a great potential for treating various types of rare diseases, which is capable of inducing a variety of genome engineering, including gene KO/KI (knock out/ knock-in), point mutation, fragment insertion, transcriptional regulation, and so on [7,8]. Much effort is put into expanding the targeting scope, improving editing efficiency, and reducing off-target frequency. The delivery system is also critical for efficient gene editing in targeted cells and individuals. According to different delivery destinations (in vivo or in vitro), the types of delivered cargo, safety concerns, and strategies, including physical delivery, non-viral chemical vector, viral vector, and virus-like particles, are discussed. We review the current progress in CRISPR-based tools for rare disease therapy with a focus on different editing strategies and delivery systems. Based on these diverse CRISPR-based toolsets, we further summarize the current therapeutic approaches in cells and animal models for fighting different rare diseases, such as blood disorders, eye diseases, metabolic disorders, and neuromuscular diseases, which aim to transfer the proof-of-concept laboratory research to clinical trials and therapeutic outcomes.

## 2. CRISPR-Based Tools for Genome Engineering

Discovered as the bacterial adaptive immune system defending against the invading genome, the CRISPR system has revolutionized our capability for performing precise genome manipulation. Compared to the previous nucleases, including meganucleases, zinc-finger nucleases (ZFN), and transcription activator-like effector nucleases (TALEN), for gene editing, the CRISPR and CRISPR-Associated (Cas) protein system provides a simple and programmable RNA-guided DNA- or RNA-editing platform [9,10,11,12,13,14]. Naturally-occurring CRISPR-Cas systems are generally divided into two classes: Class 1 (type-I, III, and IV) containing multiple protein effectors and Class 2 (type-II, V, and VI) containing a single protein effector [15]. Among them, Type-II Cas9 and type-V Cas12a nucleases are commonly used as RNA-guided DNA-targeting editing tools, while type-VI Cas13 derivatives provide RNA-targeting engineering capability.

The Cas9 nuclease has two catalytic domains named HNH and RuvC for targeting strand and non-targeting strand cleavage, respectively [16]. Mutations at one or both of the domains result in Cas9 nickase (nCas9) or catalytically dead Cas9 (dCas9), respectively [13]. Despite the impaired catalytic activity, nCas9 and dCas9 sustain their DNA-targeting property and provide a platform for genome engineering without creating double-strand breaks (DSBs), including base editors (BEs) and prime editors (PEs) [17,18,19]. The mechanism and engineering of these CRISPR-based tools are briefly introduced in this section of the review.

### 2.1. Cas Nucleases-Mediated Genome Editing

Cas nucleases induce DSBs at targeted loci, which can be repaired via endogenous DNA repair pathways, such as non-homologous end joining (NHEJ) or homology-directed repair (HDR). The NHEJ pathway can lead to the knockout of the target gene as it frequently generates indels that can create frame-shift and premature stop codons, etc. Homology-directed repair (HDR) occurs at a relatively lower frequency when donor DNA is present and can generate the desired knock-in sequence at the target locus [20,21].

**Figure 1:**
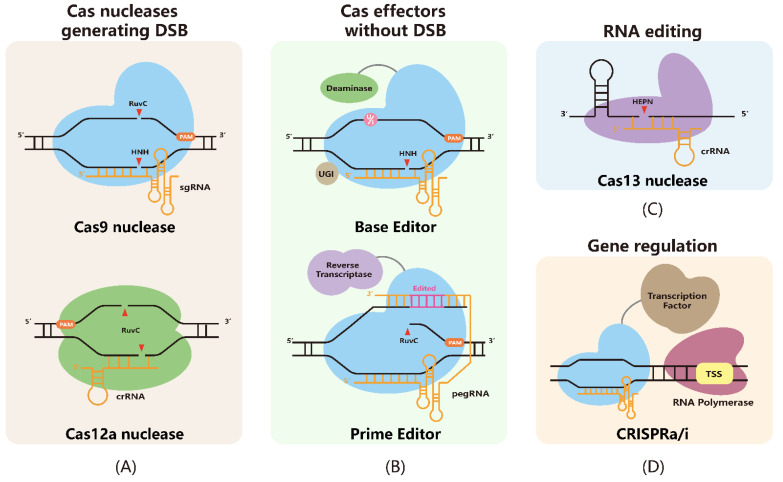
CRISPR-based tools for different engineering applications. (**A**) Cas nucleases, including Cas9 nuclease and Cas12a nuclease, generate DSBs (double-strand breaks) at targeted loci. After binding with sgRNAs (single guide RNAs) or crRNAs (CRISPR RNAs), Cas nucleases recognize the target DNA sequence adjacent to the PAM (protospacer adjacent motif) sequence and cut the DNA target. (**B**) BEs (base editors) and PEs (prime editors) achieve sequence engineering without generating DSBs. BEs use deaminases to deaminate C or A into U or I, respectively. Accordingly, subsequent DNA repair and replication process transit the deaminated nucleotides into desired T or G. PEs use 3′-extended pegRNA (prime editing guide RNA) to install the desired sequence into the target locus. These effectors use nCas9s to nick the target DNA for higher editing efficiency. (**C**) Cas13 nucleases work with crRNAs to target and cleave RNAs. Notably, different members of the Cas13 family have different crRNA orientations (not shown). (**D**) CRISPR a/i (activation/interference) effectors recruit transcription factors to dCas9 systems for targeted gene regulation without altering the DNA sequence.

The Cas nuclease is directed to its genomic target by a guide RNA, which contains a spacer region that pairs with the target DNA sequence. SpCas9 (from *Streptococcus pyogenes*) is the most commonly used Cas9 nuclease (Figure 1A). The Cas9 gRNA occurs naturally as a dual guide RNA system composed of a CRISPR RNA (crRNA) and a paired trans-activating crRNA (tracrRNA) [22]. To facilitate its ease in manipulation, a single guide RNA (sgRNA) is engineered by fusing crRNA with tracrRNA [13]. Cas nuclease recognizes DNA sequences adjacent to the protospacer adjacent motif (PAM). SpCas9 features the NGG PAM sequence, which presents at the 3′ end of the non-targeting strand and a cleavage site 3-nt upstream of PAM [23].

Other Cas effectors, such as Cas12a, *Staphylococcus aureus* Cas9 (SaCas9), CasX, CasΦ, CasMINI, and others, have also been applied for genome engineering [24,25,26,27,28,29]. For example, Cas12a is another well-developed CRISPR-based tool, characterized by the ability of crRNA self-processing and T-rich PAM sequence (Figure 1A) [30,31,32]. Cas12a only requires crRNA, which consists of repeat sequence and spacer sequence, for functional DNA cleavage [24]. Cas12a has only one RuvC-like domain that generates staggered cut distal to 5′ PAM sequence [24]. With the crRNA self-processing activity, the Cas12a enables simultaneous targeting of multiple genes with a single crRNA array and has the potential to be applied for rare diseases’ treatment. Moreover, Cas effectors with a smaller protein size can facilitate the delivery of Cas effectors for rare disease gene therapy [25,26,27,28]. These proteins differ in editing efficiency, protein size, sgRNA structure, PAM sequence, and so on, which broadens the choices of Cas nuclease effectors for editing events with different requirements.

Many efforts have been devoted to optimizing the Cas nucleases for comprehensive application, such as broadening the PAM sequence, improving HDR efficiency, and reducing off-target. For example, directed evolution and rational design have been undertaken to loosen or remodel PAM patterns by protein structure engineering, which broadens the list of editable genes [8,33]. Moreover, different strategies are introduced to maximize HDR efficiency for desired sequence changing [34]. For example, rather than inducing DSBs, nCas9 induces single-strand cleavage, which specifically stimulates HDR for higher substitution efficiency [35]. Meanwhile, cell cycle synchronization, reagents regulating HDR-related pathways, and editing at HDR-related genes increase HDR frequencies at different levels [36]. Cas9/RecA system recruits bacterial recombinase A during the editing process to increase HDR efficiency [37]. Off-target frequency can be reduced by optimizing DNA specificity and minimizing undesired DSB generation. Other than the rational design of spacer sequence with higher on-target activity, chemical modification on the sgRNA backbone shows improvement in DNA specificity [38,39]. In addition, the structure-based rational design of Cas9 variants restricts off-target frequency in the mechanism of inactive conformation being present at off-target loci [40,41,42]. A dual nCas9 system requires paired editing at the targeted locus and reduces the frequency of undesired DSBs [43]. Although reduced Cas9 expression level also decreases the off-target frequency, reduction in on-target efficiency can occur at the same time [44].

### 2.2. Base Editors-Mediated Genome Editing

While Cas nuclease-mediated genome editing is successful in different systems, Cas nuclease induces DSBs at target loci, which are repaired by natural DNA repair pathways that lead to various unpredictable repair outcomes. The HDR efficiency is usually lower than NHEJ, which can lead to random indels, insertion of unwanted sequences into the genome, and even a large deletion of genome fragments [36]. To overcome these problems, tools, such as base editors, that install precise mutations without generating DSBs are needed for fighting rare diseases.

BEs refer to a class of CRISPR-Cas-based tools engineered to install precise point mutations at the target locus (Figure 1B). Such technologies use nCas9 (D10A) to target the desired region, and fused ssDNA deaminase catalytically deaminate cytosine to uracil (e.g., CBE: cytosine base editor; TAM: targeted AID-mediated mutagenesis) or adenine to hypoxanthine (ABE: adenine base editor) [17,18,45].

Commonly used CBE fuse cytidine deaminase APOBEC (APOlipoprotein B mRNA editing enzyme, catalytic polypeptide) to the nCas9 (D10A), together with UGI (uracil DNA glycosylase inhibitor) for higher efficiency [17]. Generated uracil (U) is recognized as thymine (T) by polymerase during the DNA repair or replication process, which subsequently leads to the transition of C·G base pair to T·A base pair. ABE is fused with TadA, which is gained from an RNA adenine deaminase after direct evolution [18]. In this case, adenine (A) is deaminated into hypoxanthine (I) and is then subsequently transited to guanine (G). Other engineered tools are also proposed for base editing. For example, TAM fuses cytidine deaminase AID (activation-induced cytidine deaminase) with dCas9 for base editing efficiency.

A variety of BEs are presented for expanded editing circumstances with different requirements. Several members of the APOBEC family have been tested for editing window and base pair motif preferences [46]. Moreover, engineered deaminase based on rational design and direct evolution makes it possible to characterize BE with desired editing preferences [47,48]. The editing efficiency of BE is also a critical consideration. As uracil after cytosine deamination is subjected to uracil N-glycosylase (UNG) that removes uracil from DNA, an additional UGI is introduced for higher editing efficiency, which presents BE4 [49]. A subsequent modification of nuclear localization signal (NLS) and codon usage gives birth to BE4max and ABEmax with even higher efficiency [50]. Meanwhile, the off-target frequency can be minimized by using the Cas domain with higher DNA specificity and a reduced BE expression level [40].

In addition, ideas of CGBE (cytosine to guanine base editors) have been introduced [51,52]. The mechanism shows that after the deamination of cytosine to uracil, the BER (base excision repair) system potentially introduces guanine at the locus after uracil is repaired from DNA. After examining several BER-related proteins, rXRCC1-fused CGBE was recommended. Moreover, Td-CGBE uses TadA-8e-derived deaminase for C·G to G·C transversion [53].

Off-target efficiency in both DNA and RNA levels is one of the major concerns for the application of the BE (especially CBE) systems. DNA-based off-targets occur in a Cas-dependent manner or Cas-independent manner. The Cas-dependent off-target is caused by those Cas effectors and sgRNAs targeting the undesired regions sharing sequence similarity with targeted regions, while the Cas-independent off-targets are generated by random deamination of transient nucleotides by expression of deaminase [54,55]. To avoid these off-target effects, engineered deaminases and high-fidelity Cas effectors have been used for optimizing BE systems, such as eA3A-BE3 and Sniper ABE7.10 [47,56,57]. Furthermore, various deaminases were characterized for higher on-target efficiency and reduced off-target frequency, including RrA3F, AmAPOBEC1, and SsAPOBEC3B, etc. [46]. Other deaminase variant-derived BEs, such as YE-BE4, Td-CBE, and TadCBEs, etc., have comparable editing efficiency to conventional BEs and lower off-target frequencies [53,58,59].

RNA-based off-targets also happen in a Cas-independent manner as the BEs were derived from RNA deaminases [60,61]. Although RNA-based off-targets are transient and happen in a relatively low frequency, transcriptome-wide off-targets increase the instability and unpredictability of the BE system. The rational design of the ABE deaminase domains has been shown to reduce RNA off-targets [62]. APOBEC1 mutant-based SECURE-BE3 (SElective Curbing of Unwanted RNA Editing) and miniABEmax-based SECURE-ABE are shown to minimize transcriptome-wide RNA off-target activity [61,63]. Furthermore, the deletion of R153 within the TadA/TadA* domain shows the ability to minimize RNA off-targets in different ABE systems [64]. CE-8e-dV has been introduced with undetectable DNA or RNA off-targets in hESCs [65].

### 2.3. Prime Editors-Mediated Genome Editing

Prime editors are capable of installing different sequence editing without creating DSBs, such as point mutations (transitions and transversions), short sequence insertions, and deletions (Figure 1B) [19]. The PE system contains an nCas9 (H840A) domain that nicks the PAM sequence strand, a prime editing guide RNA (pegRNA) component, and the reverse transcriptase (RT) effector. Notably, pegRNA has a spacer sequence at the 5′ end for specific targeting and an extended 3′ end sequence as the prime binding site (PBS) and RT template for the desired editing. The generated 5′ flap containing the unedited sequence is excised during the DNA repair process, allowing the 3′ flap containing the edited sequence to be incorporated into the genome.

PE-related technologies have the potential for various gene editing occasions. Improvement of the system, however, is still needed for further therapeutic applications, such as delivery strategies and higher editing efficiency [66]. Cas-independent off-target is not detected in PE systems in mammalian cells; the editing efficiency of PEs is less robust than BEs [67]. There are several versions of PEs for higher editing efficiency and lower off-target frequency [19]. For example, appending a structured RNA motif to the 3′ end of pegRNA prevents it from degradation and subsequently enhances editing efficiency [68]. PEs have also been applied for the insertion and deletion of large DNA sequences [66]. The PRIME-Del technology induces the deletion of large DNA sequences up to 10 kb by using a pair of pegRNA, targeting the opposite strands of DNA, flanking the targeted region [69]. Furthermore, the twinPE is capable of integration (>5 kb) and inversion (>40 kb) of large DNA fragments using paired pegRNA combined with serine recombinase Bxb1 [70]. The homologous 3′ extension-mediated prime editor (HOPE) uses paired pegRNA to target both strands with higher editing efficiency and less off-target activity [71].

### 2.4. CRISPR-Cas13-Mediated RNA Editing

The CRISPR-Cas13 system provides RNA-guided RNA nucleases, containing the HEPN (higher eukaryotes and prokaryotes nucleotide-binding) domain and the RNase activity domain (Figure 1C). The Cas13 family is classified into Cas13a, Cas13b, Cas13c, Cas13d, Cas13X, and Cas13Y, etc. based on crRNA structure and protein size [72]. After binding with the crRNA, Cas13 undergoes a conformational change and performs RNA-guided ssRNA degradation. Notably, in addition to on-target RNA editing, activated Cas13 shows a collateral effect, which cleaves surrounding non-specific RNAs once activated [73]. This feature has been developed for the detection of nucleic acid in vitro, such as SHERLOCK (Specific High-sensitivity Enzymatic Reporter Unlocking) [74]. Although the collateral effect is generally undetectable in mammalian cells, some evidence suggests it can restrict the therapeutic application in certain cases [75]. The screening of engineered Cas13d and Cas13X variants identified candidates with minimal collateral effect and similar editing efficiency, compared to wild-type proteins [76].

Cas13-based base editors have broadened the application of RNA therapy. REPAIR (RNA Editing for Programmable A-to-I Replacement) system induces adenosine to inosine deamination by fusing ADAR2 adenosine deaminase [77]. By fusing a directionally evolved ADAR2 cytidine deaminase, the RESCUE (RNA Editing for Specific C-to-U Exchange) system induces cytosine to uracil deamination [78]. A subsequent substitution of the Cas13 domain to ultrasmall Cas13bt pushes the therapeutic application of Cas13-based base editors even further due to the ease of in vivo delivery [79].

Cas13-mediated RNA therapy does not induce permanent genome mutation. On the one hand, such manipulation has the advantage regarding safety concerns [80]. On the other hand, it requires constant administration of Cas13 effectors for lasting ease of symptoms. Moreover, the transient nature of RNA editing is suitable for targeting diseases related to temporal changes in cell state [72,77]. RNA editing reversibly and controllably manipulates transcriptomes, making the CRISPR-Cas13-based tools increasingly popular for fighting rare diseases [72].

### 2.5. CIRSPRa/i-Mediated Transcription Regulation

CRISPR activation/interference systems (a/i) fuse transcription factors (TFs), for example, VPR (VP64-p65-Rta) for activation and KRAB (Krüppel associated box) for interference, to dCas9 for transcription regulation at targeted sites (Figure 1D) [81,82]. Fused TFs recruit or repress RNA synthesis at TSS (transcription start sites) for downstream transcription regulation. SunTag-based CRISPRa system recruits multiple copies of antibody-fused VP64 for enhanced activation at the targeted locus [83]. Moreover, genome-scale CRISPRa/i libraries are established and characterized for mapping complex in vivo pathways [84]. CRISPRa/i has the potential for therapeutic application as it does not alter the genome sequence. Such applications require a long-term expression of the CRISPRa/i system during therapy for stable manipulation.

## 3. Delivery System of CRISPR-Based Tool

**Figure 2:**
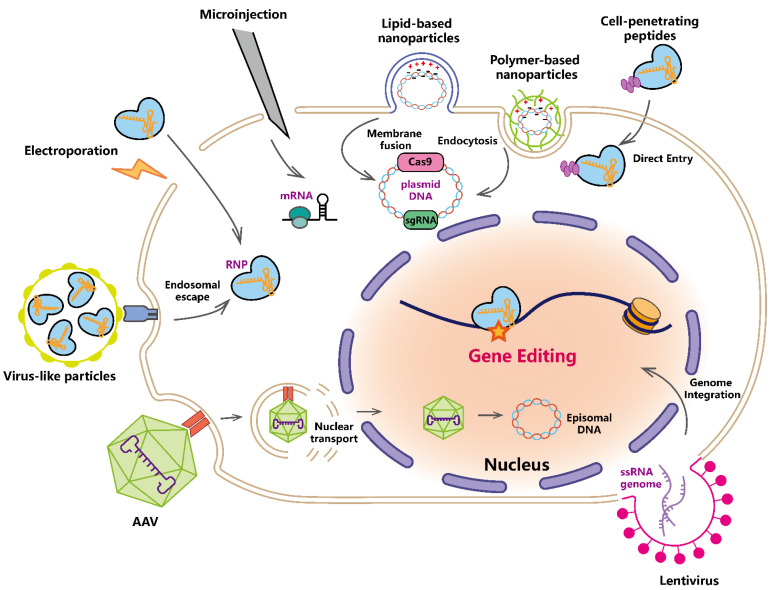
Delivery systems for CRISPR-Cas cargos. Physical delivery uses electroporation and microinjection to directly deliver CRISPR systems in the forms of plasmid DNA, mRNA, or RNP (ribonucleoprotein) into cells. Positively charged lipid-based nanoparticles and polymer-based nanoparticles transfer the encapsulated negatively charged cargos into cells through membrane fusion and endocytosis. Cell-penetrating peptides mediate the direct entry of fused RNPs into cells. Lentivirus and AAV (adeno-associated virus) are commonly used viral delivery systems. The lentivirus undergoes reverse transcription and integrates into the genome for subsequent transcription. AAV undergoes endosomal escape and the released genome transports into the nucleus and remains as episomal DNA. Virus-like particles can transiently deliver RNPs into cells with a quick onset time. Delivered or translated Cas effectors mediate gene editing at target loci in the nucleus. Please note that delivered cargos and the mechanism of cell entry are not fully presented for each delivery system.

To apply the CRISPR-based tool into therapeutic practice, the delivery system is critical for efficient editing. The CRISPR system can be delivered ex vivo into isolated cells or in vivo to living organisms, respectively [85]. Meanwhile, it can be delivered in different forms: (1) DNA encoding Cas effector and sgRNA; (2) mRNA encoding Cas effector and expressed sgRNA; (3) RNP (ribonucleoprotein) complex consisting of Cas effector and sgRNA [86]. DNA delivery features a long active time, slow onset time, and requires genome integration in certain scenarios. Although mRNA and sgRNA have relatively short onset time and median active time, this type of delivery is costly and less stable. RNP delivery takes effect the most quickly and is transient for lower off-target mutations [87]. Delivery strategies include physical delivery, non-viral chemical delivery, viral vectors, and virus-like particles (VLP) etc. (Figure 2).

### 3.1. Physical Delivery

Examples of physical delivery strategies include electroporation and microinjection (Figure 2) [87]. Electroporation increases cell membrane permeability with pulses of electric current to transfer CRISPR-Cas cargos into target cells [88]. Microinjection uses micro-scale needles to inject CRISPR components into desired subcellular locations [89]. High delivery efficiency can be achieved with these strategies for different forms of CRISPR systems (DNA, mRNA, or RNP). However, they are generally restricted to ex vivo delivery for various restraints. A strong electric field damages cell viability during electroporation, while microinjection is laborious and requires operational experience [87]. Conditions for physical delivery need to be adjusted according to the cell type and other experimental variables for optimal performance.

### 3.2. Non-Viral Chemical Delivery

Non-viral chemical delivery uses chemical-based nanoparticles or peptides to facilitate the transfer of CRISPR-Cas cargos. Lipid-based nanoparticles (LNP) are commonly used chemical vectors (Figure 2) [90]. Negatively charged DNA, mRNA, and RNP are encapsulated into the positively charged liposome in an aqueous environment due to electrostatic interaction and hydrophobic interaction. LNPs subsequently fuse with lipid bilayers of the cell membrane and release the contents. Lipofectamine-based LNP delivery has been used for both in vivo and in vitro gene editing [91,92]. Meanwhile, efforts have been done to clarify the immunological and toxicological concerns of liposomes, such as interactions with cell membrane proteins for opsonization and dysfunctional effect on organs [93].

Alternatively, non-lipid polymer-based nanoparticles, including polyethylenimine and poly-L-lysine, are also commonly used for delivery (Figure 2) [85]. Similarly, positively charged polymer-based nanoparticles encapsulate negatively charged cargos and are subjected to cell endocytosis for release. Compared to other delivery system, chemical nanoparticles are not specific to certain cell types and suffer from low efficiency, which limits the therapeutic application of these methods.

In addition, the CRISPR-Gold system uses gold nanoparticles (AuNPs) as delivery media for immune response-free in vivo delivery due to its chemical inertness [94,95]. AuNPs are conjugated to the 5′-thiol of donor DNAs, which are then ligated to RNPs and encapsulated into the polymer PASp(DET) particles [94]. Although the technology shows the potential to be applied to in vivo delivery, further attempts and modifications are needed.

Cell-penetrating peptides (CPP) are short peptides with the ability to transfer across the cell membrane (Figure 2). CPP can mediate delivery by coupling to Cas effector and sgRNA components [96]. Although CPP-mediated delivery can achieve reasonable efficiency for in vitro editing, complicated biological environments restrict its usage in many in vivo applications [96].

### 3.3. Viral Delivery System

Viral delivery systems represent a class of engineered virus-based media, which contain the transfection ability while being blocked for further genome replication and viral generation in transfected cells for safety concerns [97]. Virus vectors based on lentivirus, adenovirus, and adeno-associated virus (AAV) are used in clinical trials. CRISPR-Cas system can be constructed in the engineered viral genome for functional viral packaging.

Lentivirus is an ssRNA genome-encoded spherical retrovirus (Figure 2) [98]. After the fusion of lentivirus particles to the cell membrane, the RNA-based genome undergoes reverse transcription and host cell genome integration, leading to subsequent expression of CRISPR-Cas effectors. Lentivirus vectors feature high package capacity (up to 8 kb), high transfection efficiency targeting various cells, including non-dividing cells, and mild immunogenicity [87]. The random genome integration enables long-term editing activity but also leads to increased risks of oncogenesis and unwanted disruption of cellular function [99]. A point mutation in the integrase generates integration-defective lentivirus (IDLV) with an extrachromosomally active genome. Though IDLVs are less efficient compared to unmutated lentiviruses [100], efforts have been made to optimize this technology [101].

Adenovirus is a dsDNA genome-encoded non-enveloped virus with icosahedral nucleocapsid [87,97]. After the endocytosis of adenovirus into the cell, the viral capsid is disassembled from endosomal escape and releases the DNA genome for subsequent nuclear pore transport [97,102,103]. The DNA genome encoding CRISPR-Cas effectors remains predominantly extrachromosomal without insertion into the host cell genome [104]. Adenovirus vector features large capacities (up to 36 kb) and is reasonably efficient for different cell types, including non-dividing cells, but has relatively high immunogenicity [97].

AAV is an ssDNA genome-encoded dependoparvovirus, which means that it is dependent on other viruses, such as adenovirus for functional life cycles (Figure 2) [97]. Cell membrane-contacted AAV undergoes endocytosis, leading to the downstream disassembly of capsid in the endosome with low pH [105]. Released ssDNA genome transfers into the nucleus and synthesizes the complementary strand for the proper expression of encoded CRISPR-Cas effectors. Moreover, AAVs with different serotypes have been used for targeting specific types of cells [97]. Although AAV2, AAV5, and AAV8 share similarities in targeted cell types, AAV8 is superior in terms of low immunogenicity and high delivery efficiency [106]. AAV9 delivery has been widely used for targeting the brain and spinal cord due to its ability to transfer across the blood–brain barrier [107,108]. Although AAV has the advantages of the lowest immunogenicity and stable transgene expression, the limited capacity (<4.7 kb) draws concerns for efficient delivery. Using a smaller Cas nuclease can ease the problem, but the restrictive PAM sequence and low editing efficacy often make it inapplicable [109]. Various strategies have been proposed for higher AAV capacity, such as using two complementary genomes, homologs recombination of vectors, and intein-mediated protein splicing [110,111,112]. For example, the engineered split-PE system enables the efficient in vivo delivery of oversize PE effectors by dual-AAV [113]. Compared to others, viral delivery systems are highly efficient for in vivo and in vitro delivery. Safety concerns, on the other hand, should be addressed in terms of immunogenicity and off-target mutations.

### 3.4. Virus-like Particles (VLPs)

VLPs are a class of delivery vectors that self-assemble viral envelopes and structural proteins into particles without the viral genome (Figure 2) [114]. By co-transfecting plasmids that encode Gag-fused CRISPR-Cas effector and other viral proteins, such as VSV-G (envelope glycoprotein of the vesicular stomatitis virus) and protease, into package cells, viral envelope-coated particles self-assemble with Gag-fused CRISPR-Cas effectors. The co-expressing of sgRNA during the process leads to preloaded RNP in VLPs [115]. In destination cells, after being cleaved from Gag proteins, CRISPR-Cas effectors are released for genome engineering [116].

Nonetheless, the integrity of the Cas effector can be affected if protease cleavage sites are presented during the assembly [117]. NanoMEDIC bypasses the protease cleavage process by co-expressing VSV-G-FKBP12 and Cas9-FRB protein [118]. Chemical-induced dimerization of FKBP12 and FRB domains leads to the loading of Cas9 into VLPs [118]. Furthermore, the eVLP (engineered virus-like particles) system uses different coated glycoproteins to realize cell type-specific targeting delivery of BE and Cas9 RNP [119]. The SEND (Selective Endogenous eNcapsidation for cellular Delivery) system uses PEG10, a mammalian Gag homolog that packages its mRNA, to facilitate the delivery of cargo mRNA [106].

VLP shows tremendous potential in ex vivo and in vivo delivery of CRISPR-based tools due to its low immunogenicity, high engineering capacity, and reduced off-target effects due to transient exposure of RNP or Cas effector-encoding mRNA. Yet, VLP-based delivery is hindered by low package yield and editing efficiency [120]. Improvement and application of VLP delivery are expected in the near future for rare disease therapeutics.

## 4. Applications of CRISPR Tools in Rare Disease Therapy

CRISPR-based techniques have opened up new possibilities for treating rare diseases. Rare diseases are often early onset, chronic, and severe, leading to early death or lifelong disability, and many of them are monogenic diseases [121]. To date, a small number of orphan designations have been authorized for some rare diseases. However, the majority of rare diseases (about 95%) lack effective treatment [122]. CRISPR-based engineering tools have been used for rare disease treatment by ex vivo or in vivo delivery systems (Figure 2), bringing a new possibility for a permanent cure with a single dose (Table 1). Here, we are going to elaborate on the gene therapy strategies for treating rare diseases based on the CRISPR engineering tools, focusing on blood disorders, eye diseases, metabolic diseases, and neuromuscular diseases.

### 4.1. Blood Disorders

The hematologic system plays a prominent part in the body’s most critical functions. For example, the hemoglobin within red blood cells is responsible for the delivery of oxygen to all the tissues throughout the body. Sickle-cell disease (SCD) and β-thalassemia are the most common inherited blood diseases in the world [123]. Both diseases are monogenic diseases caused by mutations of the hemoglobin β subunit gene (*HBB*), which leads to variations in the β-globin chain. Moreover, they are representative disease models that use the ex vivo gene-editing strategy; the patient’s blood stem cells can be harvested from their blood for ex vivo editing and then put back into their bloodstream. The ex vivo strategy is safer and more effective, given that ex vivo gene editing ensures that CRISPR tools only perform functions in the target cells.

To date, two types of CRISPR-based gene therapy approaches have been developed for the treatment of SCD and β-thalassemia. One approach is to correct the mutated base directly using base editors. This method can convert the pathogenic codon (GTG) to a non-pathogenic codon (GCG), leading to the transformation from the HbS (valine) to the HbG-Makassar (alanine). Recently, Newby et al. used ABE8e-NRCH to treat SCD by converting a targeted A⋅T base pair into G⋅C in hematopoietic stem and progenitor cells (HSPCs) from SCD patients with high efficiency (68%) and no observed off-target mutations [124]. To overcome the PAM limitation of potential base editing targets, Beam Therapeutics Inc. (Cambridge, MA, USA) designed a set of inlaid base editors (IBEs) by inserting deaminase (TadA*) in different locations of Cas9, allowing editing of the HbS locus (BEAM-102) with 80% efficiency [125]. These results demonstrated that the base editors are highly efficient at treating blood disorders.

Except for directly targeting the mutated genes, an alternative approach is to restore fetal hemoglobin (HbF) expression for SCD and β-thalassemia treatment. Previous studies have demonstrated that the *BCL11A* gene encodes a transcription factor that represses γ-globin subunits expression and HbF in erythroid cells [126,127]. Meanwhile, studies have shown that certain mutation at the −198 position of the promotor region of the γ-globin genes *HBG1* and *HBG2* (referred to as the “British mutation”) leads to the hereditary persistence of HbF into adulthood [128]. Based on the findings, researchers have tested that the γ-globin protein can be reproduced via breaking the BCL11A erythroid-specific enhancer, disrupting the *BCL11A* binding site (CCAAT Box) in the *HBG* gene or creating the “British mutation”.

**Table 1:** Applications of CRISPR-based tools for fighting rare diseases.

Diseases Type	Diseases	Gene	Strategy	Animal Model or Cells	Delivery	Outcome	References; Clinical Trials
Blood disorder	SCD/b-thalassemia	*HBB*	ABE8e-NRCH-mediated point mutation correction	HSPCs from patients with SCD	RNP; electroporation	80% editing efficiency, 72% decrease in the pathogenic protein	[124]
		*HBB*	IBE-mediated point mutation correction	CD34^+^ HSPCs/erythroid differentiated cells	mRNA; electroporation	77% editing efficiency, 80% Makassar editing	[125] Clinical trials
		*HBG1*, *HBG2*	ABE8s-mediated point mutation to create “British mutation” and increase levels of γ-globin	CD34^+^ cells, human T cells	RNP; electroporation	80% editing efficiency, 60% protein knockdown efficiency	[129] Clinical trials
		*HBB* (−28)	A3A(N57Q)-BE3-mediated BCL11A enhancer disruption to reproduce γ-globin	CD34^+^ HSPCs	RNP; electroporation	>20% editing efficiency	[47]
		*BCL11A* (+58)	A3A(N57Q)-BE3-mediated BCL11A erythroid-specific enhancer disruption to reproduce γ-globin	CD34^+^ HSPCs	RNP; electroporation	93.3% editing efficiency, restoring >60% of γ-globin	[130]
		*BCL11A*	NHEJ-mediated BCL11A erythroid-specific enhancer disruption	CD34^+^ HSPCs	RNP; electroporation	80% editing efficiency, 30% decrease in sickle hemoglobin	[131] Clinical trials
		*HBB*	NHEJ-mediated mRNA splicing	CD34^+^ HSPCs	Cas12a RNP; electroporation	>30% editing efficiency, restoring >60% of γ-globin	[132]
		*BCL11A*	NHEJ-mediated BCL11A erythroid-specific enhancer disruption	HSPCs	AsCas12a/Cpf1 RNP; electroporation	~80% editing efficiency	[133]
		*HBG*	NHEJ-mediated HBG disruption	CD34+ cells	AsCas12a/Cpf1 RNP; electroporation	>80% editing efficiency	[134] Clinical trials
Eye diseases	LCA10	*CEP290*	HDR-mediated IVS26 mutation deletion	HEK293FT/Mice	Dual AAV5	7.5–26.4% editing efficiency	[135]
		*CEP290*	NHEJ-mediated IVS26 mutation deletion	iPSC	AAV5	>50% editing efficiency	[136]
		*Cep290*	HDR-mediated IVS26 mutation deletion or inversion	Mice	AAV5	~30% editing efficiency	[137] Clinical trials
	LCA2	*Rpe65*	HDR-mediated point mutation correction	Mice	AAV9	>1% editing efficiency	[138]
		*Sox2*, *Klf4* or *Oct4*	HyperdCas12a activator-mediated endogenous transcription factor gene activation	Mice	In vivo electroporation		[139]
Metabolic disorders	FH	*LDLR*	HDR-mediated base insertion	iPSC	Cas9 RNP; electroporation	~10% editing efficiency	[140]
		*Ldlr*	HDR-mediated point mutation correction	Mice	Single AAV8	6.7% editing efficiency, restoring 18% of LDLR protein	[141]
		*Pcsk9*	Intein-mediated SaCBE-mediated point mutation correction	Mice	Dual AAV8	25% editing efficiency	[142]
		*PCSK9*	ABE8.8-mediated mRNA splicing	Macaca fascicularis	LNP	90% PCSK9 reduction	[143,144] Clinical trials
		*Pcsk9*	SaKKH-ABE8e-mediated mRNA splicing	Mice	Single AAV8	66% editing efficiency, 93% PCSK9 knockdown efficiency	[145]
		*Pcsk9*	Split-cPE573-mediated stop codon insertion	Mice	Dual AAV8	13.5% editing efficiency	[146]
		*Pcsk9*	BE3-mediated stop codon generation	Mice (in utero)	Adenoviral (Ad) vectors	14.5% editing efficiency	[147]
		*Pcsk9*	ABE-eVLPs-mediated the splice donor disruption for Pcsk9 knockdown	Mice	eVLPs	63% editing efficiency	[119]
	HT1	*Fah*	HDR-mediated point mutation correction	Adult Mice	Hydrodynamic injection	0.4% editing efficiency, restoring 8–36% of Fah mRNA	[148]
		*Fah*	HDR-mediated point mutation correction	Adult Mice	Lipid Nanoparticles (Cas9 mRNA) and AAV (sgRNA/HDR)	6% editing efficiency, restoring 9.5% of Fah mRNA	[149]
		*Fah*	Intein-split BE4max-mediated point mutation correction	Mice	Dual rAAV	31% editing efficiency	[150]
		*Hpd*	NHEJ-mediated gene deletion	Mice	Hydrodynamic tail vein injection	The editing efficiencies at 1 and 4 weeks were 8% and 68%	[151]
		*Hpd*	BE3-mediated stop codon generation	Mice	Adenoviral (Ad) vectors	36% editing efficiency	[147]
	PKU	*Pah*	Intein-split nSaKKH-BE3-mediated point mutation correction	Mice	Dual AAV8	25.1% editing efficiency, restoring 63% of PAH mRNA	[152]
Neuromuscular diseases	DMD	*Dmd*	Split-intein NG-ABEmax-mediated exon 50 skipping	Mice	Dual AAV9	35% editing efficiency, restoring 54% of dystrophin protein expression	[153]
		*DMD*	PE2-mediated two bases insertion	iPSC-derived cardiomyocytes	P3 Primary Cell 4D-Nucleofector X Kit	54% editing efficiency, restoring 39.7% of dystrophin protein expression	[153]
		*Dmd*	ABE7.10-mediated point mutation correction	Mice	Trans-splicing AAV	3.3% editing efficiency, restoring 17% of dystrophin protein expression	[154]
		*Dmd*	SaCas9-mediated exon 23 deletion	Neonatal Mice	AAV9	39% editing efficiency, restoring 7% of dystrophin protein expression	[155]
		*DMD*	TwinPE-mediated exon 51 deletion	HEK293T	Lipofectamine 2000	28% editing efficiency	[70]
		*Dmd*	NHEJ-mediated mRNA (exon 50) splicing	Mice	AAV9	27.9% editing efficiency, restoring 90% of dystrophin protein expression	[156]
		*DMD*	KKH SaCas9-based TAM-mediated exon 50 skipping	iPSC	Lipofectamine LTX Reagent	90% editing efficiency	[157]
		*Dmd*	CRISPR-Cas9-AID (eTAM)-mediated exon 4 skipping	Mice	AAV9	>50% editing efficiency, restoring ~90% of dystrophin protein expression	[158]
		*Dmd*	ADAR-mediated point mutation correction	Mice	AAV8	3.6% editing efficiency, restoring 1–2.5% of dystrophin protein expression	[159]
	ALS	*SOD1*	HDR-mediated point mutation correction	iPSCs	Plasmid; electroporation	20% editing efficiency	[160]
		*FUS*	HDR-mediated point mutation correction	iPSCs	Plasmid; electroporation	1% editing efficiency	[160]
		*C9orf72*	HDR-mediated MRE correction	iPSCs	Plasmid; electroporation	0.6–4.5% editing efficiency	[161]
		*C9orf72*	HDR-mediated MRE correction	iPSCs	Plasmid; electroporation	Reducing 22.5% of C9orf72 MRE expression	[162]
	DM1	*DMPK*	NHEJ-mediated MRE excision	myoblasts	Nucleofection	51% editing efficiency	[163]
		*DMPK*	NHEJ-mediated MRE excision	Fibroblast/myoblasts	RNP; electroporation	14% editing efficiency	[164]
		*DMPK*	NHEJ-mediated MRE excision	DM1 iPSCs	Nucleofection	>50% editing efficiency	[165]
		*DMPK*	NHEJ-mediated MRE excision	iPSCs and myogenic cells	Lentivirus	>50% editing efficiency	[166]
		*DMPK*	PIN-dRCas9 mediated CUG repeat RNA cleavage	myoblasts and fibroblasts	AAV9/Lentivirus		[167]
		*Dmpk*	PIN-dRCas9 mediated CUG repeat RNA cleavage	Neonatal mice/Adult mice	AAV9		[168]
		*DMPK*	LshCas13a-mediated CUG repeat RNA cleavage	myoblasts	Lentivirus	20% reduction in DMPK mRNA level	[169]
	NPC	*Npc*	BE3-mediated point mutation correction	Mice	AAV9	59% editing efficiency	[142]

Accordingly, CRISPR Therapeutics (Basel, Switzerland) and Vertex Pharmaceuticals (Boston, USA) disrupted the region of *BCL11A* enhancer with CRISPR-Cas9-mediated NHEJ to restore γ-globin expression, and nearly 80% of the alleles were modified at this locus without observed off-target editing (CTX001) [131]. AsCas12a-mediated NHEJ is also applied for SCD and β-thalassemia therapy by targeting the *BCL11A* or *HBG*-binding site, with high editing efficiency and HbF induction [133]. Due to different cutting mechanisms, Cas12a-mediated NHEJ can induce a larger deletion (>3 bp) than Cas9, resulting in robust HbF expression. Although they can achieve high editing efficiency and induce HbF expression, both systems create DSB, leading to unpredictable DNA repair outcomes and a high possibility of off-target effects.

In addition, researchers employed CBE to destroy the *BCL11A* enhancer, preventing sickling and ameliorated globin subunit imbalance in erythroid cells that are seen in SCD and β-thalassemia [47,130]. Beam Therapeutics Inc. used ABE8s to achieve A·T to G·C conversion at HBG1/2 promoters for restoring the HbF expression, thus, mitigating the defects in β-globin that occurred in SCD and β-thalassemia (BEAM-101) [129].

### 4.2. Eye Diseases

The eye is an ideal organ for in vivo gene editing, since it is small, relatively contained, and has minimal immune reactivity. For example, leber congenital amaurosis (LCA) is an inherited retinal degenerative disease caused by mutations in different genes, including *CEP290*, *GUCY2D*, *CRB1*, and *RPE65* [170,171]. LCA is featured with childhood-onset blindness and its incidence is nearly 1 in 80,000 [172]. The most common form of LCA, Leber congenital amaurosis type 10 (LCA10), is caused by loss-of-function mutations in the centrosomal protein 290 kDa (*CEP290*) gene, which encodes the protein necessary for the assembly of the connecting cilium of photoreceptors and phototransduction [173]. The disease results in severe vision loss or blindness in early infancy. A point mutation located within intron 26 (c.2991 + 1655A>G), abbreviated as IVS26 mutation, generating a new splice donor site and creating a premature stop codon, is a common cause of LCA10 (accounting for up to 15% of all LCA cases) [174,175]. LCA2 caused by mutations in the *RPE65* gene is the second most common form of LCA, accounting for about 6% of LCA cases [170]. In recent years, studies have proved that CRISPR-Cas9 could excise or convert the IVS26 cryptic-splice mutation in iPSC and LCA10 mouse models via NHEJ and HDR pathway for the LCA10 treatment concomitantly with a pair of sgRNAs [135,136,137]. CRISPR-Cas9-mediated HDR is also used to correct the *RPE65* nonsense mutation [138].

In addition to CRISPR-mediated gene editing, the CRISPRa/i system has the potential for gene therapy applications. For example, Guo et al. employed the hyperdCas12a activator and a single crRNA array to activate three endogenous transcription factors for altering the differentiation of retinal progenitor cells in the mouse retina, providing new insights for eye disease therapy [139].

### 4.3. Metabolic Disorders

Metabolic disorders are diseases caused by disruption to the normal metabolic pathways, which are commonly caused by loss-of-function mutations in metabolic enzymes, resulting in the accumulation of toxic metabolic intermediates or the deficiency of one or more metabolites inside the body. Metabolic disorders mainly affect a few organs, including the liver, intestine, and pancreas of the body. Among them, the liver is a preferable target organ for gene therapy, and many types of AAV, such as AAV8, AAV9, and AAV-DJ, have strong cytotropism for the liver [176,177]. Therefore, based on CRISPR-mediated gene editing, researchers have developed various gene therapy approaches for treating metabolic diseases of the liver, such as familial hypercholesterolemia (FH), hepatorenal tyrosinemia type 1 (HT1), phenylketonuria (PKU), and so on. Here, we use FH and HT1 as examples to elaborate on the related gene therapy strategies.

Familial hypercholesterolemia (FH) is an autosomal co-dominant metabolic disease featured with high content of low-density lipoprotein cholesterol (LDL-C) in plasma [178,179]. The high level of circulating LDL-C in plasma increases the risk of atherosclerotic plaque development and cardiovascular disease. The disease is commonly caused by mutations in the *LDLR*, *APOB*, and *PCSK9* genes encoding the low-density lipoprotein receptor (LDLR), apolipoprotein B (APOB), and PCSK9, respectively.

LDLR is a cell membrane protein that transfers excess LDL from plasma circulation into the liver for being degraded by the lysosome, and defects in its function can result in FH. Mutations in the *LDLR* gene that lead to a low protein expression of functional LDLR are one of the main causes of FH (accounting for 90%) [180]. In 2017, CRISPR-Cas9-mediated HDR was used to insert the missing 3 bp in *LDLR* exon 4 to correct the genetic mutation and restore the LDLR expression and cholesterol metabolism [140]. Subsequently, Zhao et al. delivered the CRISPR-Cas9 to the *Ldlr^E208X^* mutant mice that carry a nonsense mutation in the exon 4 of the *Ldlr* gene by AAV8 to modify disease-causing gene mutation with an efficiency of 6.7% in vivo [141]. These results have demonstrated that CRISPR-Cas9-mediated HDR provides a promising therapeutic approach for FH treatment (Table 1).

Proprotein convertase subtilisin/kexin type 9 (PCSK9) encoded by the *PCSK9* gene is the ninth member of the protein convertase family, which can bind to the epidermal growth factor A (EGF-A) domain of the *LDLR* to induce LDLR degradation [181]. Recent studies showed that loss-of-function mutations in *PCSK9* significantly reduce the level of LDL-C in patients without adverse health effects, suggesting that PCSK9 can be safely inhibited as an effective target for FH [182,183]. Therefore, researchers have developed different CRISPR-based approaches to inhibit the PCSK9 function for FH treatment (Table 1). Rossidis et al. delivered a CRISPR base editing system (BE3) in utero to artificially introduce a stop codon in the *Pcsk9* gene through AAV9, thus, reducing the plasma PCSK9 and cholesterol [147]. In 2020, Levy and co-workers used the dual AAV-CBE3.9max constructs packaged in AAV8 with a sgRNA to create the *Pcsk9* W8X mutation in mice with 25% of editing efficiency [142]. To reduce the off-target effect of CBE, Wang et al. constructed a transformer BE, termed tBE, for C-to-T editing via the fusion of a cleavable deoxycytidine deaminase inhibitor (dCDI) with deoxycytidine deaminase (mA3CDA1) [184]. They delivered the tBE system to edit the *Pcsk9* gene in mice through dual AAV8 vectors for creating a premature stop codon in exon 4 of *Pcsk9* with 30% editing efficiency, thus, reducing PCSK9 and 30–40% of LDL-C in plasma. Moreover, the NG-ABE8e RNP is delivered to the liver of mice through eVLPs to break the splice donor for *Pcsk9* knockdown [119].

Hepatorenal tyrosinemia type 1 (HT1) is a severe hereditary disease caused by mutations in the *FAH* (fumarylacetoacetate hydrolase) gene with an incidence of 1 in 100,000 [185]. The disease results in liver failure due to the accumulation of toxic intermediate metabolites (Maleyl-acetoacetate and Fumaryl-acetoacetate) from the tyrosine metabolic pathway. Previous studies have reported that HT1 could be ameliorated in adult *Fah*−/− mice through CRISPR-Cas9-mediated HDR gene editing [148,149]. Yang et al. developed an intein-split BE4max to efficiently induce C-to-T conversion in an HT1 mouse model harboring a start codon mutation in the *Fah* gene through rAAV vectors [150]. This method achieved 31% of the desired conversion to generate a start codon and restored FAH expression without the observed off-target mutations (Table 1).

In addition, deleting the upstream 4-hydroxyphenylpyruvate dioxygenase (HPD) encoded by *HPD* in the tyrosine metabolic pathway can prevent the accumulation of the toxic intermediate metabolites without other adverse effects [185]. Based on this, 2-(2-nitro-4-trifluoro-methylbenzoyl)-1,3 cyclohexanedione (NTBC), a small molecule drug, has been applied for HT1 treatment [186]. In 2016, Pankowicz et al. treated HT1 in mice by deleting the *Hpd* gene using CRISPR-Cas9 [151]. Rossidis and co-workers administered Ad-BE3 with an sgRNA targeting *Hpd* to generate a nonsense mutation, thus, decreasing the HPD level and rescuing the lethal phenotype [147]. Therefore, the CRISPR tools have brought new ways for treating metabolic diseases.

### 4.4. Neuromuscular Disease

Neuromuscular diseases are a class of disorders that lead to the injury or dysfunction of the peripheral nervous system or skeletal muscle, including Duchenne muscular dystrophy (DMD) [187], amyotrophic lateral sclerosis (ALS) [188], myotonic dystrophy type I (DM1) [189], and Niemann-Pick type C disease (NPC) [190]. A variety of CRISPR-based strategies are developed for gene therapy of neuromuscular diseases, including CRISPR-Cas9-mediated NHEJ and HDR, base editors, prime editors, RNA-targeting Cas9, and Cas13 system. Next, we will focus on the related gene therapy methods for neuromuscular disease using DMD treatment as an example.

DMD is the most prevalent fatal X-linked, progressive neuromuscular degenerative disease, causing the skeletal muscles’ dystrophy and later respiratory or heart failure [187]. DMD is caused by frameshifting mutations or nonsense mutations in the *DMD* gene that result in premature truncation of the encoded dystrophin protein. In comparison, Becker muscular dystrophy (BMD) is a milder neuromuscular disease caused by mutations in the DMD gene that maintains the open reading frame, producing internally deleted but partially functional dystrophin proteins [191]. Therefore, compared with DMD, the symptom onset of BMD is late and the progression is slow. This comparison provides a theoretical base for DMD treatment using micro-dystrophin and exon-skipping strategies.

In recent years, many efforts have been made to rescue the biological function of dystrophin protein in vivo through gene therapy, including AAV-mediated delivery of gene-encoding micro-dystrophin [191,192,193], correction of nonsense mutation with base editors, deletion of an exon or base insertion to restore the reading frame by CRISPR-Cas9-mediated genome editing and prime editing (PE) [153], exon-skipping strategies to “skip over” mutation-harboring or mutation-adjacent exons in the *DMD* mRNA using antisense oligonucleotides (ASO), and CRISPR-Cas9-mediated genome editing and base editors [156,157,158,194] (Table 1).

There are several CRISPR strategies being developed to restore the reading frame of the *DMD* gene to reproduce shorter but functional dystrophin protein. Mutated *DMD* exon could be excised to produce in-frame mRNA and restore dystrophin expression in the mouse model of DMD via multiple CRISPR-based technologies, such as CRISPR-Cas9-mediated NHEJ and PE [70,155]. Meanwhile, Chemello et al. applied the PE strategy to insert two bases in exon 52 to reconstruct the reading frame for the treatment of DMD patients with the exon 51 deletion mutation, which could restore nearly 40% of dystrophin protein [153]. Notably, approximately 4% of normal dystrophin expression is sufficient for improving muscle function [195]. Ryu et al. used ABE7.10 to correct a nonsense mutation in the exon 20 of the *Dmd* gene in a mouse model with DMD, restoring 17% of dystrophin expression without obvious unwanted indels and off-target mutations [154].

In addition, based on the principle of splicing machinery, researchers have capitalized on CRISPR tools to correct the splice sites, causing exon skipping and restoring the reading frame of the *DMD* transcript. In 2017, Amoasii et al. made reframing mutations in the splicing acceptor of exon 51 in the *Dmd* mouse model without exon 50 via AAV delivery, allowing the skipping of exon 51. This approach achieved 7.9% of editing efficiency at the targeted genomic site and restored up to 90% of dystrophin protein expression [156]. With the emergence of base editors, researchers exploited base editors to create mutations at the splicing sites of targeted exons separately, resulting in targeted exon skipping and restoring the reading frame. Both methods reached >35% of editing efficiency and generated functional dystrophin proteins [45,153,157,158].

In addition, researchers have developed several RNA editing methods to achieve gene therapy. For example, an AAV9-packaged RNA-targeting Cas9 (RCas9) system targeting CUG repeats has been developed to degrade the toxic RNA and reverse DM1-associated aberrant splicing in patient myotubes and HSA_LR_ mouse models with high efficiency and specificity [167,168]. CRISPR-Cas13 with CUG-flanking guide RNA has been used to cleave CUG repeat RNA and eliminate toxic tandem microsatellite expansion RNA in the DM1-patient-derived myoblast cell line via lentiviral delivery [169]. In addition, Katrekar et al. engineered ADAR guide RNAs to manipulate ADARs for editing the stop codon in exon 23 of the *DMD* transcript [159]. Together, different methods have been developed for the treatment of neuromuscular diseases at the level of DNA or RNA using CRISPR-Cas9 or CRISPR-Cas13 (Table 1).

## 5. Discussion and Future Directions

CRISPR-based engineering tools are widely used for genome editing and manipulation of gene expression, opening up new pathways for rare disease therapy. Targeted insertion, deletion, or base conversion of the gene could be obtained using different CRISPR engineering tools. For example, the targeted insertion or deletion of genomic sequences can be achieved by CRISPR-Cas-mediated NHEJ or HDR pathways and prime editors. Point mutation correction can be achieved by base editors and prime editors that mediate base transition and transversion. Base editors can also be used to introduce a stop codon through base transition for the gene knockdown. Together, these tools can meet the need for the treatment of many rare diseases. It is possible to cure a rare disease with a single dose via CRISPR-mediated DNA editing. In addition to DNA editing, RNA editing also holds some advantages in therapeutic applications, since it is reversible and tunable without bringing about permanent changes in the genome. RNA editing tools (for example, REPAIR or RESCUE) are proposed for safe and efficient editing at the RNA level, which holds great promise for treating diseases caused by abnormal alternative splicing [77,78].

However, there are still many challenges for clinical application, which requires further improvement and optimization of CRISPR-based engineering tools. For example, it is necessary to develop CRISPR engineering tools with small size, high efficiency, and low off-target. In addition, as for point mutation correction, there is a dire need for the base editor to only target an adenine or cytosine within the editing activity window.

Although many delivery systems are already applied to deliver CRISPR plasmids, mRNAs, and RNPs into targeted cells or tissues for DNA or RNA editing ex vivo or in vivo, there are still many challenges regarding safety, payload capacity, and targeting specificity. AAV vectors are generally considered as a relatively safe and efficient delivery system and are widely used to deliver CRISPR nucleases into specific tissue. However, the safety of the rAAV delivery system is still a concern and needs to be carefully monitored in clinical application. In recent years, highly engineerable VLP and extracellular nanovesicles are gradually leveraged to deliver RNP into cells or tissues [118,119,196]. However, the content of VLPs or extracellular nanovesicles packaged from the producer cells is unclear, and future work needs to address potential immune responses in the targeted cells [119]. Future efforts to improve safety, specificity, and editing efficiencies in different tissues can further broaden the therapeutic potentials of VLPs and extracellular nanovesicles. Considering the large number of rare diseases, only a small portion of rare diseases are being tested for treatment by the existing CRISPR tools. Although these tools open a new approach for the treatment of rare diseases, it is necessary to improve both the efficiency and safety of the current toolbox and delivery system for expanded usage.

In addition, CRISPR-based regulation and epigenetic engineering tools (for example, CRISPRa and CRISPRi) manipulate the level of gene expression without altering DNA sequences. As early as 2017, Ho S-M et al. reported that the level of endogenous neuropsychiatric risk gene expression could be manipulated in human iPSCs-derived neural progenitor cells, neurons, and astrocytes by applying CRISPRa/i, suggesting the potential of CRISPRa/i system for treating psychiatric disorders [197]. Recently, Thompson et al. used CRISPRa screening to identify genes and pathways that can upregulate type VII collagen (C7) expression to seek the targeted drug for dystrophic epidermolysis bullosa [198]. Although several compounds have been used to increase C7 expression, CRISPRa has shown excellent potential for disease treatment. Other epigenetic editing strategies using dCas recruitment of epigenetic effectors (for example, p300/CBP, HDAC3, DNMT3A, or TET1) have been proposed for the locus-specific acetylation, deacetylation, methylation, or demethylation, which can be expected for the therapeutics of inherited diseases caused by epigenetic changes (for example, fragile X syndrome) [199,200,201,202,203,204].

Many trials for rare diseases using CRISPR-based engineering tools are still in their early stages. The majority of existing strategies tend to break a gene, rather than repair a gene, largely because of the difficulty in the insertion or deletion of large DNA sequences. In addition, current CRISPR-based engineering tools mostly target monogenetic diseases. Multiplex genome editing tools are necessary for polygenetic disease treatment. Yuan Q. et al. have developed a multiplex base- and prime-editing strategy to simultaneously edit multiple genomic loci (up to 31 loci) in human cells, which shows the potential for the treatment of polygenetic diseases [205]. Altogether, the improvement of CRISPR-based engineering tools and delivery systems will provide promising avenues for fighting rare diseases in the future.
